# Restoration of Degraded Grassland Significantly Improves Water Storage in Alpine Grasslands in the Qinghai-Tibet Plateau

**DOI:** 10.3389/fpls.2021.778656

**Published:** 2021-12-16

**Authors:** Xiaowei Guo, Huakun Zhou, Licong Dai, Jing Li, Fawei Zhang, Yikang Li, Li Lin, Qian Li, Dawen Qian, Bo Fan, Yuting Lan, Mengke Si, Bencuo Li, Guangmin Cao, Yangong Du, Bin Wang

**Affiliations:** ^1^Key Laboratory of Adaptation and Evolution of Plateau Biota, Northwest Institute of Plateau Biology, Chinese Academy of Sciences, Xining, China; ^2^College of Ecology and Environment, Hainan University, Haikou, China; ^3^New South Wales Department of Primary Industries, Wagga Wagga Agricultural Institute, Wagga Wagga, NSW, Australia

**Keywords:** alpine grasslands, degraded grassland restoration, grass litter, Qinghai-Tibet plateau, soil-water storage

## Abstract

Alpine grassland has very important water conservation function. Grassland degradation seriously affects the water conservation function; moreover, there is little understanding of the change of water state during grassland restoration. Our study aims to bridge this gap and improve our understanding of changes in soil moisture during the restoration process. In this study, the water storage, vegetation, and meteorology of a non-degradation grassland (grazing intensity of 7.5 sheep/ha) and a severely degraded grassland (grazing intensity of 12–18 sheep/ha) were monitored in the Qinghai-Tibet Plateau for seven consecutive years. We used correlation, stepwise regression, and the boosted regression trees (BRT) model analyses, five environmental factors were considered to be the most important factors affecting water storage. The severely degraded grassland recovered by light grazing treatment for 7 years, with increases in biomass, litter, and vegetation cover, and a soil-water storage capacity 41.9% higher in 2018 compared to that in 2012. This increase in soil-water storage was primarily due to the increase in surface soil moisture content. The key factors that influenced water storage were listed in a decreasing order: air temperature, litter, soil heat flux, precipitation, and wind speed. Their percentage contributions to soil-water storage were 50.52, 24.02, 10.86, 7.82, and 6.77%, respectively. Current and future climate change threatens soil-water conservation in alpine grasslands; however, grassland restoration is an effective solution to improve the soil-water retention capacity in degraded grassland soils.

## Introduction

The Qinghai-Tibet Plateau (QTP) plays a critical role in regional sustainable development and water resource conservation. This plateau is home to many rivers in China and Southeast Asia, and plays a key role in ensuring water security (Wang and Fu, 2004). In this region, the alpine grasslands contribute significantly to water conservation *via* the water retention. The alpine grassland covers an area of 1.5 × 10^8^ ha and accounts for 50.9% of the total area of the QTP. Therefore, alpine meadow is the most representative vegetation on the QTP. However, 30 % of the alpine grasslands are now severely degraded, due to the combined influence of climate change and human disturbance (Dai et al., 2021). Since the 1970s, grassland degeneration of the QTP has been recognized by many researchers, policy makers, and managers (Dong et al., 2020), degeneration severely threatens ecological security at regional and global scales. Additionally, the field surveys of grassland vegetation carried out at the beginning of this century demonstrated the alpine grasslands have indeed degraded, compared with those in the 1980s (Yang et al., 2006). Between 1992 and 2002, grassland degradation in the central and northwestern of the QTP was very serious, and degradation is more severe at high altitudes (4300–4600 m above sea level; Fassnacht et al., 2015). The degradation of plateau vegetation has a severe impact not only on China, but also global ecological security.

The water conservation function of alpine grassland is strongly affected by climate change and overgrazing. Evapotranspiration in grassland ecosystems is being continuously enhanced due to global warming, which reduces the water conservation capacity of grassland soil (Guo et al., 2020). However, the impact of climate change on grassland water conservation is slow and indirect. Conversely, the impact of grazing is not, and grazing can lead to serious grassland degradation within only three to 5 years (Mipam et al., 2019). Grassland degradation is the main reason for reducing the soil-water conservation function of grassland on the QTP (Dai et al., 2020). Even in the serious degraded grasslands that have been reclaimed into artificial grasslands, there is no significant improvement in water conservation function (Shen et al., 2020). The soil saturated moisture capacity, the capillary moisture capacity, and the field moisture capacity increase under light grazing, while these values drop sharply under heavy grazing. The saturation point and field capacity in moderately degraded grasslands increase by 17.1 and 5.8%, respectively, compared to less degraded grasslands (Zhao et al., 2011). However, the saturated moisture capacity, capillary moisture capacity, and field moisture capacity of the grassland decrease sharply in severely or extremely degraded grasslands (Kruemmelbein et al., 2009). The soil-water conservation function in degraded grassland has reduced by 18.3–27.8% on the QTP (Jiang et al., 2016), and therefore, moderate grazing of alpine grasslands is paramount to maintaining the water conservation function of alpine grassland.

Previous studies on grassland degradation and its effects on water conservation function have predominantly focused on spatial, rather than temporal data coverage; for example, comparing the water conservation function of grasslands with different degrees of degradation across different sites. However, alpine grassland vegetation and soil conditions vary geographically (Wang et al., 2016), and preclude complete explanations for changes in soil-water storage capacity. Our study monitored the same site of degraded alpine grassland for a period of 7 years, while the meadow was being restored through reduced grazing pressure, and postponing autumn grazing to give the seeds time to mature. The collected long-term dataset can be used to investigate the factors that are responsible for changes in soil-water capacity. The restoration of degraded grasslands can improve the vegetation condition, soil particle size, soil macro-aggregates, and organic matter content of the grassland, and these attributes in turn can lead to an improvement in the soil-water storage. Based on seven consecutive years of grassland moisture monitoring, we hypothesize that the restoration of degraded grasslands in the QTP will act to increase their soil-water storage capacity. We propose that the main reason for this change is the change of vegetation characteristics.

## Materials and Methods

### Site Description and Experimental Design

This study was carried out in the Haibei National Field Research Station in the alpine grassland ecosystem (N37.6109°, E101.3142°, 3200 m above sea level), located in the northeastern region of the QTP. Seasonally, frozen ground is well developed in this region, which is characterized by a plateau continental monsoon climate. The mean annual air temperature is −1.70°C and the yearly precipitation is 570 mm, 80% of which occurs from May to September (Chen et al., 2008). The vegetation type is typical alpine meadow.

Our study observed the soil moisture content in the two plots selected during the growing seasons from 2012 to 2018. Two flat winter grassland plots along were selected, one with a native grassland (NG; N37.6109°, E101.3142°, 3212 m above sea level) and were another with a severely degraded grassland (SDG; N37.7003°, E101.5828°, 3268 m above sea level). The two plots were 2.8 km apart. They have been lightly grazed since 2012, with a grazing intensity of 7.5 sheep/ha, the difference between the two plots was caused by different previous grazing pressures. The SDG plot owned by a farmer was heavily grazed for 10 years with a rough estimate grazing intensity about 12–18 sheep/ha. Conversely, the SDG plot was gradually restored, the NG plot was operated by the Haibei National Field Research Station, and its grazing intensity had never changed. Due to a reduction in the grazing intensity from 2012 to 2018, detailed information about the two plots was provided in Table 1. Based on our field investigation, the soil in both plots was classified as Mat Cry-gelic Cambisols, which is rich in organic matter in the surface layer, because some plant roots in alpine grassland have surface accumulation. There are a lot of dead roots in the soil surface. The QTP has a relatively cold climate and the decomposition of organic matter is relatively slow, so the soil surface organic matter is relatively high.

**Table 1:** Characteristics in the plots studied from 2012 to 2018.

Year	2012		2018	
Site	NG^†^	SDG	NG	SDG
Exposed soil coverage (%)	1 ± 1.0^‡^	21.8 ± 5.9	1 ± 1.0	8.9 ± 3.2
Total biomass (gm^−2^)	183.6 ± 32.8	4.4 ± 2.2	181.6 ± 22.9	126.5 ± 31.6
Organic matter (g/kg)	162.1 ± 22.1	145.8 ± 8.7	189.8 ± 34.9	158.6 ± 28.7
total nitrogen (g/kg)	7.6 ± 0.7	6.9 ± 0.4	8.6 ± 1.2	8.1 ± 0.9
Available phosphorus (mg/kg)	9.1 ± 0.4	7.4 ± 1.0	15.7 ± 1.6	12.8 ± 1.4
Nitrate nitrogen (mg/kg)	12.8 ± 2.2	5.4 ± 1.4	18.8 ± 3.7	17.8 ± 4.8
Ammoniacal nitrogen (mg/kg)	13.4 ± 1.7	12.7 ± 3.8	16.9 ± 3.0	15.5 ± 3.0
Dominant species composition and plant community description	Two-layer canopy. Upper canopy: *Stipa aliena*, *Helictotrichon tibeticum*, and *Elymus nutans*. Lower canopy: *Kobresia humilis, Scirpus distigmaticus, Poa crymophila*, and *Dracocephalum heterophyllum*	One-layer-canopy. *Elsholtzia calycocarpa, Ajania tenuifolia, Polygonum sibiricum, Ligularia virgaurea, and Potentilla anserine*.	Two-layer canopy. Upper canopy: *Stipa aliena, Helictotrichon tibeticum*, and *Elymus nutans*. Lower canopy: *Kobresia humilis, Scirpus distigmaticus, Poa crymophila*, and *Dracocephalum heterophyllum*	Two-layer canopy. Upper canopy: *Stipa aliena, Helictotrichon tibeticum*, and Elymus nutans. Lower canopy: *Kobresia humilis, Scirpus distigmaticus, Poa crymophila*, and *Dracocephalum heterophyllum*

† NG, native grassland; SCG, severely degraded grassland.

‡ Mean ± SE of three replicates were presented.

### Data Collection

**Figure 1:**
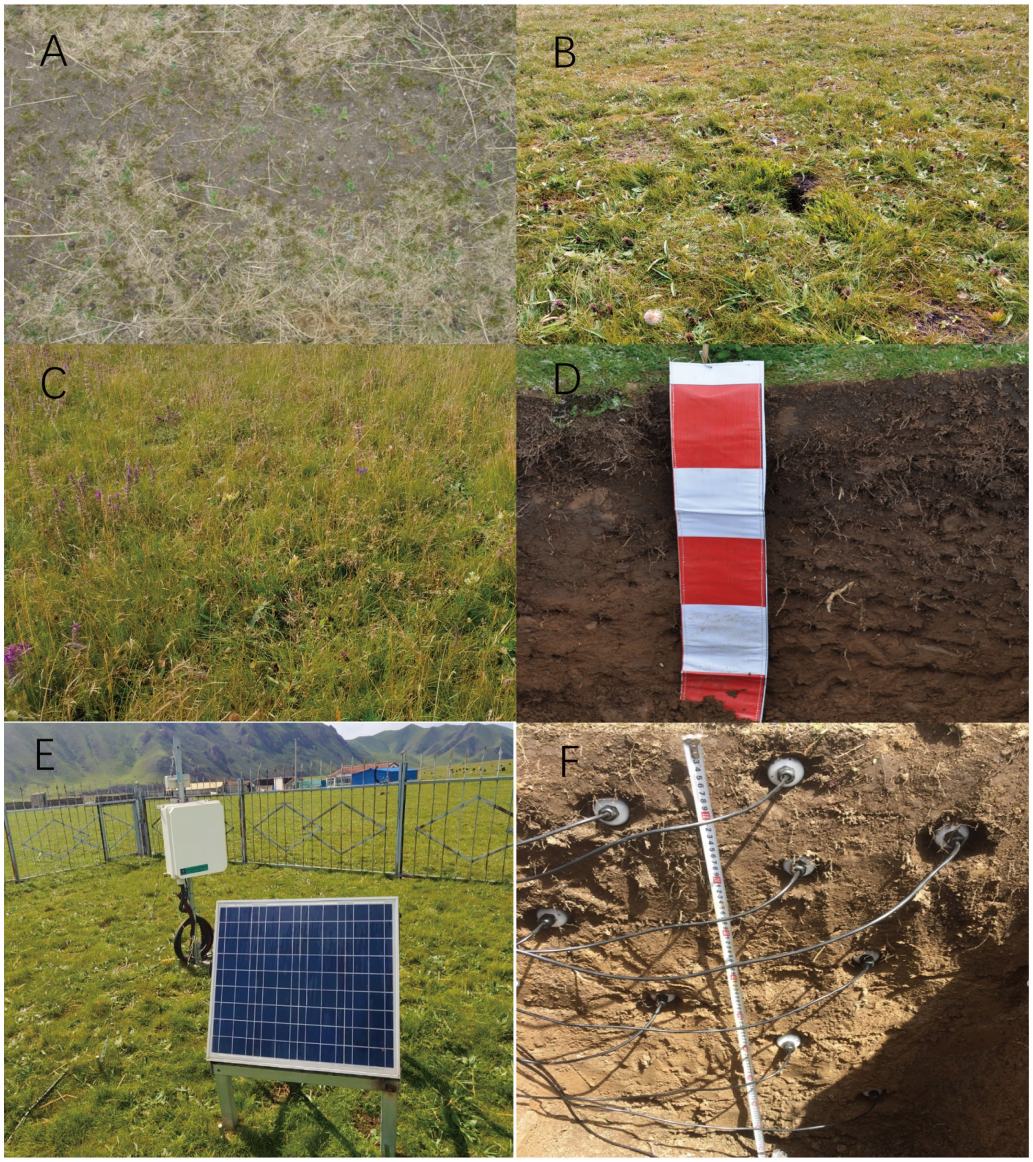
The experiment plots and the soil moisture monitoring system. ^*^Surface vegetation photograph of SDG in 2008 **(A)** and 2018 **(B)**, Surface vegetation photograph of NG in 2018 **(C)**, the Soil profile photograph **(D)**, the soil moisture monitoring system **(E)** and Hydra Probe **(F)**.

Daily meteorological data including relative humidity, wind speed, net radiation, soil temperature, and mean air temperature were obtained from a meteorological station between 2012 and 2018. Precipitation data were obtained by collecting precipitation manually. Soil moisture was measured from 2012 to 2014 using the drying and weighing method, oven drying for 24 h, then calculating the difference between dry weight and wet weight. Soil moisture was automatically measured by the soil monitoring system (A755; Campbell, United States; Figure 1). The probe type of soil moisture automatic observation system is Hydra Probe (America, Campbell), which was set at the soil depth of 5 cm, 10 cm, 15 cm, 20 cm, 30 cm, and 50 cm. This probe can quickly and continuously measure soil moisture, temperature, and electrical conductivity every hour. Time Domain Reflectometry (TDR) is the principle of soil moisture measurement in this study, because the dielectric constant of liquid is much higher than soil. The dielectric constants were calculated from the pulse velocity, and the relationship between dielectric constant and soil-water storage could be described by a third-order polynomial (Nadler, 1991; White et al., 1994).

The plant community was surveyed every month in the growing season using a grid method of quadrat survey procedure (0.5 m × 0.5 m). Vegetation coverage was calculated based on the occurrence within the 100 points. The above-ground biomass and grass litter were obtained by a standard harvesting method, then quickly brought back to the laboratory and dried at 75°C for 24 h before weighing.

Daily soil-water storage, expressed as mm, was defined as the actual soil-water content in a soil sample of a given thickness under natural conditions, is calculated as follow (Guo et al., 2020):

W=F×H×SD×10 ( 1 )

where W is the soil-water storage (mm), F is the bulk density of soil (g/cm^3^), H is the thickness of the soil (cm), and SD is the soil moisture content (%).

### Statistical Analysis

The significance of differences in soil-water storage, soil moisture content, and soil temperature between the different soil layers was analyzed using ANOVA and POST-HOC tests (LSD method). All differences were tested for significance at *p* < 0.05 level with SPSS 20.0 (SPSS Inc., Chicago, IL, United States). There are some disadvantages to examine the individual effects of environmental variables on soil-water storage solely based on the coefficients of independent variables, in a multiple regression analysis, which cannot split the individual contribution rate of each factor. The boosted regression trees (BRT) model was adopted to quantitatively evaluate the relative influence of environmental variables on soil-water storage. The BRT model was conducted with R software version 4.1.1.

## Results

### Soil-Water Storage in the Two Plots

**Figure 2:**
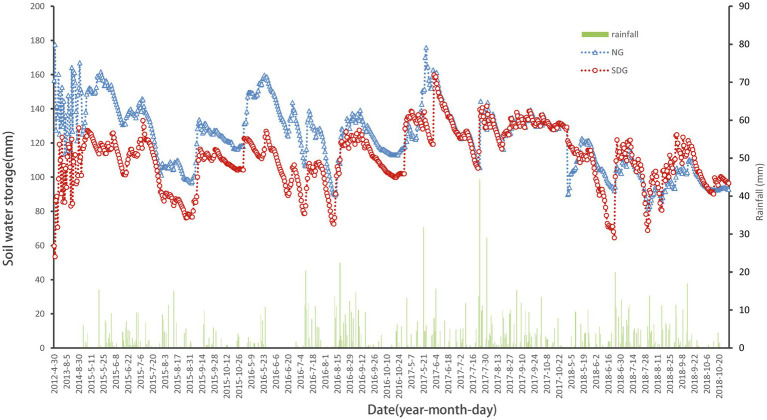
Changes in grassland water storage during the growing season. ^*^Due to the lack of data, the precipitation data began on May 1, 2014.

From 2012 to 2018, there was no significant change in the soil-water storage capacity of NG. However, the soil-water storage capacity of the degraded grassland plot increased every year due to the decrease in grazing pressure. The soil-water storage in the degraded grassland plot in 2017 and 2018 was significantly higher than in 2012 and 2013 (*p* < 0.05; Figure 2); in 2018, it was 41.9% higher than in 2012. The annual difference of soil-water storage was significant from 2012 to 2018 (*p* < 0.05), especially in the degraded grassland plot. The soil-water storage in NG was significantly higher than that in the degraded grassland plot in the years 2012, 2013, 2014, and 2015 (*p* < 0.05) but showed no significant differences in the years 2016, 2017, and 2018. Soil-water storage in the growing season changed periodically every year, with a double peak (mid-May and mid- to late-August) and single valley observed in each change curve. The minimum value usually occurred in mid-August.

### Changes in the Environmental Characteristics at the SDG Plot

**Figure 3:**
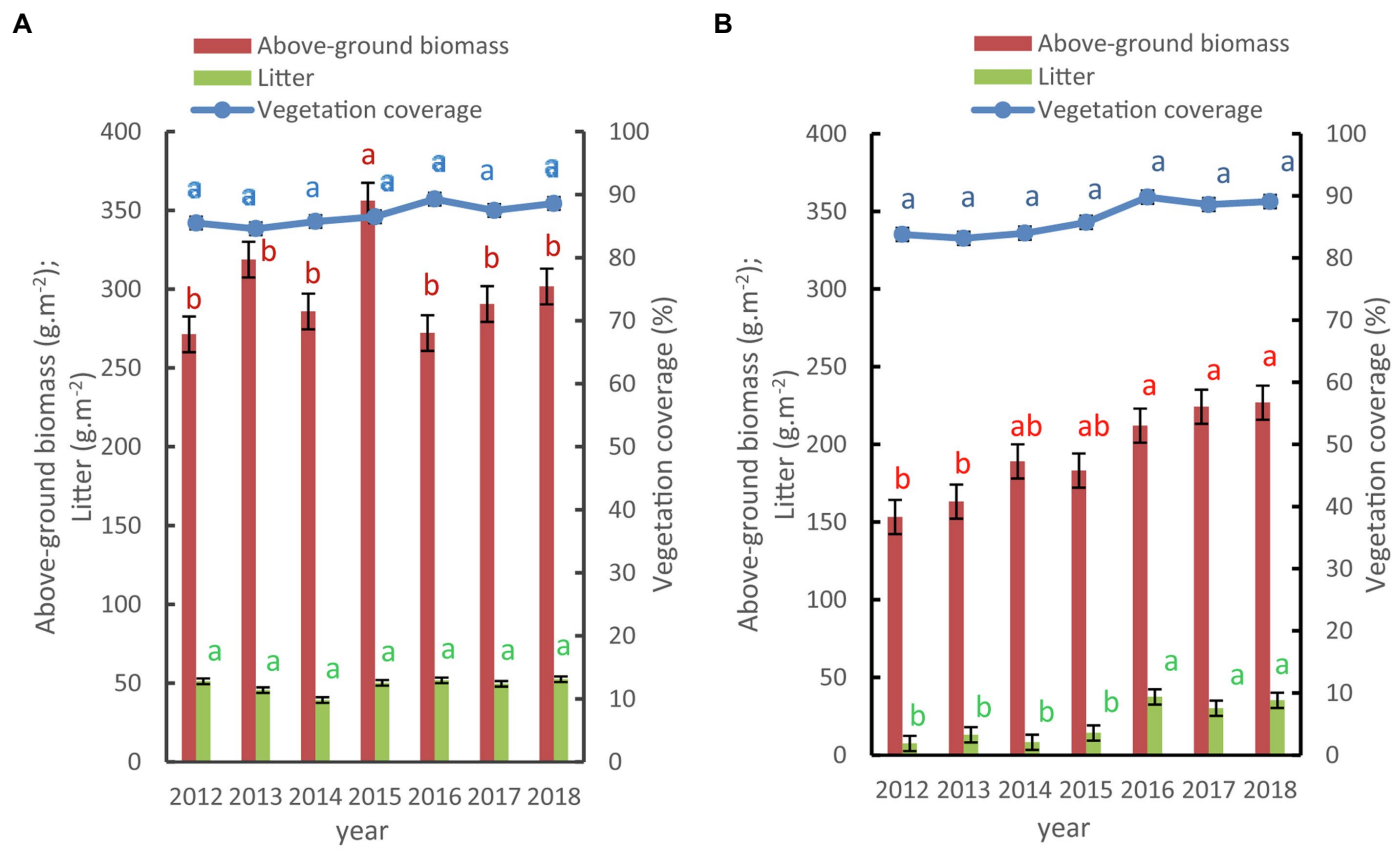
Changes in the vegetation characteristics of the severely degraded grassland plot. ^*^vegetation characteristic from 2012 to 2018 at NG **(A)** and SDG **(B)**.

The SDG plot was gradually restored from 2012 to 2018 due to the reduction of grazing pressure. The degenerative state of this area improved from a severe to light degradation status over this time, and its above-ground biomass, vegetation cover, and litter showed increasing trends (Figure 3). The above-ground biomass increased by 38.4% in 2018 compared to that in 2012. The bulk density of degraded grassland was higher than that of native grassland in each soil layer (Table 2).

**Table 2:** The soil bulk density of NG and SDG in 2018.

Year	Soil bulk density in NG (g/cm^3^)	Soil bulk density in SDG (g/cm^3^)
0–10 cm	0.71 ± 0.05	0.75 ± 0.01
10–20 cm	0.86 ± 0.06	0.94 ± 0.08
20–30 cm	0.95 ± 0.04	1.13 ± 0.06
30–40 cm	1.19 ± 0.07	1.41 ± 0.11
40–50 cm	1.44 ± 0.01	1.47 ± 0.04

A one-way ANOVA and POST-HOC tests (LSD method) showed that the biomass in the degraded grassland in 2018 was significantly higher than that in 2012 and 2013 (*p* < 0.05). The biomass increased year by year in 2016, 2017, and 2018, but it did not reach a significant level. Also, the vegetation covers of the degraded alpine grassland increased and was 83.8 ± 4.2%, 83.2 ± 6.9%, 84 ± 5.3%, 85.7 ± 6.2%, 89.8 ± 5.1%, 88.6 ± 4.7%, and 89.1 ± 3.9%, respectively, from 2012 to 2018. However, the ANOVA results showed that there was no significant difference in vegetation cover between these years. The amount of litter was the vegetation parameter that showed the largest variation. The litter density in the degraded grassland in 2016, 2017, and 2018 was significantly higher than that in 2012 and 2013 (*p <* 0.05) by POST-HOC tests (LSD method). The density of litter in 2018 had increased 3.7 times than that in 2012.

**Figure 4:**
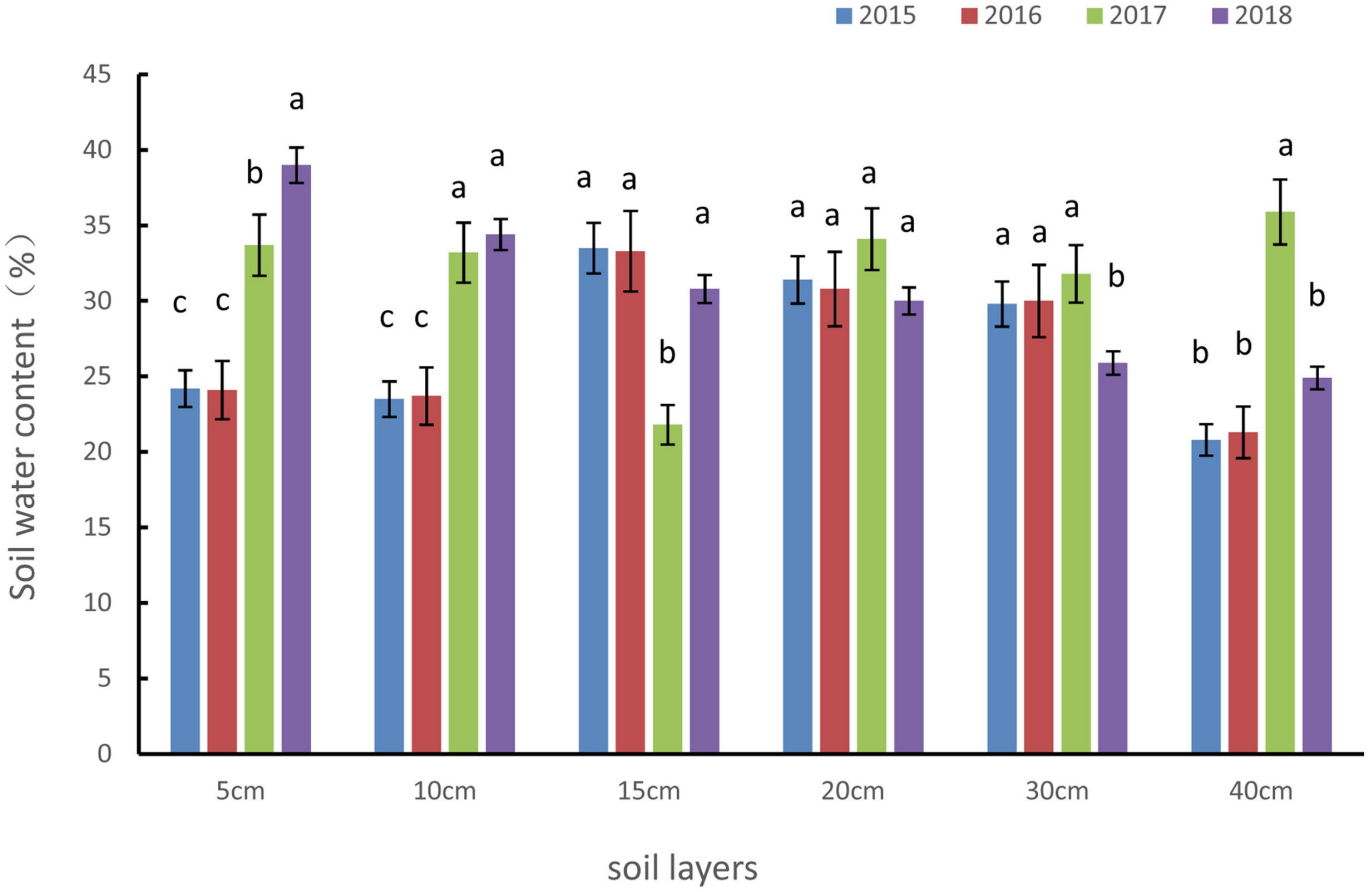
Changes in the soil moisture content at different soil depths in the severely degraded grassland.

Soil-water storage in SDG increased every year. We compared the soil moisture content at the same soil depths from 2015 to 2018 and found that the main reason for the increase in soil-water storage was an increase in surface soil moisture content (Figure 4). The POST-HOC tests (LSD method) showed that the soil moisture content at the 5 cm and 10 cm depths increased significantly (*p* < 0.01), while the soil moisture at the 30 cm and 40 cm depths did not show any obvious changes. The soil moisture content in 5 cm and 10 cm increased by 61.2 and 46.3%, respectively, in 2018 compared with 2015.

We wanted to identify the key factors affecting soil-water storage in alpine grasslands. We conducted a correlation analysis among soil-water storage and 16 environmental factors including above-ground biomass, vegetation cover, litter, root biomass, wind speed, air temperature, humidity, atmospheric pressure, precipitation, total radiation, reflected radiation, ultraviolet radiation, net radiation, effective radiation, and soil heat flux. The results showed that wind speed, precipitation, underground, and litter showed a positive correlation with soil-water storage (*p* < 0.05). Conversely, air temperature, net radiation, soil heat flux, and vegetation cover were negatively correlated with soil-water storage (*p* < 0.05).

A stepwise regression analysis and a dominance analysis were conducted to further understand how these eight factors jointly affect soil-water storage. The stepwise regression equation obtained was as follows:

Soil-water storage = 0.048 L +0.439 precipitation +2.247 wind speed – 2.409 air temperature + 5.679 soil heat flux (*r*^2^ = 0.176, *p* < 0.01).

**Figure 5:**
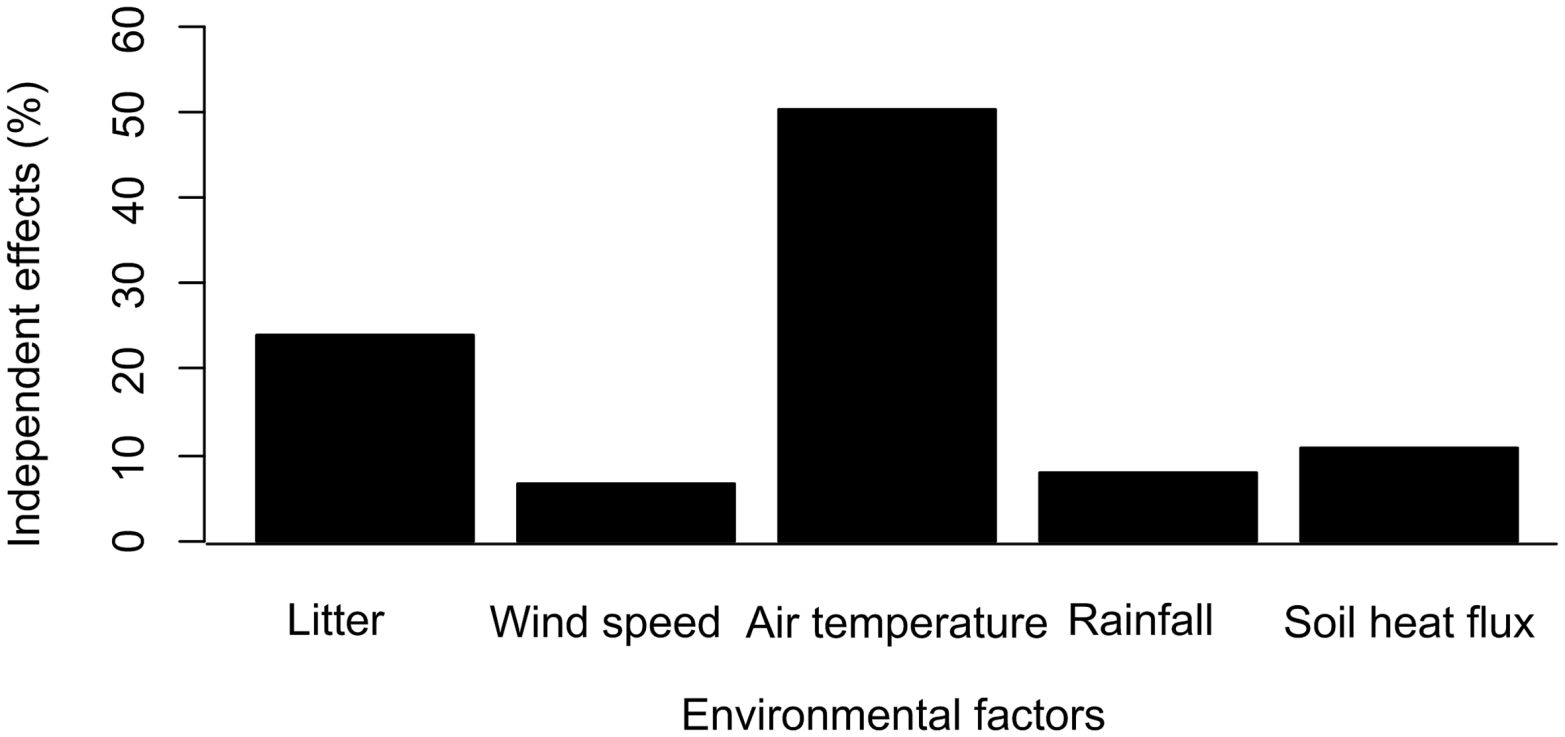
Independent effects of environmental factors on soil-water storage.

We conducted a dominance analysis using the BRT model, and the top five important factors that influenced water storage were listed in a decreasing order: air temperature, litter, soil heat flux, precipitation, and wind speed (Figure 5). Their relative contributions to soil-water storage were 50.52, 24.02, 10.86, 7.82, and 6.77%, respectively. Based on the BRT model analysis, we found that the role of climate factors in determining soil-water storage was greater than that of vegetation factors. Air temperature and litter together explained 74.5% of the total variation, which were two key factors controlling the soil-water storage of grassland.

## Discussion

### Soil-Water Storage in Alpine Grasslands

The soil-water storage capacity in alpine grasslands experienced obvious fluctuations during the growing season, where the typical curve illustrates two peaks and a single valley (Figure 2). The two peaks occur at mid-May and early September, coinciding with the beginning and end of the growing season, and the minimum value of soil-water storage occurs at early August. Other ecosystems generally show a fluctuation characterized by a single peak and valley (Oudin et al., 2008; Ren et al., 2019). This difference is attributed to the combined impacts of permafrost thawing, rising temperatures, and transpiration from vegetation. The soil in alpine grasslands is mostly comprised of seasonally frozen soil, which forms in early November and disappears completely by mid-May of the following year, with a maximum depth of 2 m (Zhang et al., 2019). The thawing of seasonally frozen soil in mid-May was the reason for the first peak in soil-water storage; the plant water consumption in the growing season makes the grassland water storage appear the lowest value at early August. Therefore, we imply that the effect of precipitation on soil-water storage is not as large as that of vegetation transpiration consumption. Vegetation transpiration in alpine grasslands accounts for 68.6–73.9% of the total evapotranspiration (Hongqin et al., 2018). By early September, some alpine grassland plants have withered, reducing the vegetation transpiration, and leading to a second peak in soil-water storage.

Soil-water storage showed a strong relationship with surface soil moisture content (depths at 5 cm and 10 cm), but at a depth of 40 cm, the soil moisture content did not fluctuate significantly throughout the growing season. This illustrates the dependence of soil-water storage on surface vegetation and environmental conditions. During the restoration of the degraded grassland, biomass and litter will increase to reduce soil evaporation. In our seven-year study, the soil-water storage capacity of the degraded grassland increased every year, as it was being gradually restored. Vegetation transpiration in alpine meadows during the peak of the growing season does not support soil-water storage, while moderate grazing leads to a reduction in transpiration. Therefore, reducing grazing on well-grown grasslands can protect soil moisture, and banning grazing is not considered a promising management strategy to improve the soil-water storage capacity in these grasslands. Several previous studies have shown that grazing bans can lead to an increase in plant growth and the disappearance of sedge plants. Therefore, in our study site, we recommend a grazing intensity of 8.5 sheep/ha to restore soil water.

### Factors Influencing Grassland Water Storage

Soil-water storage in a grassland is distinctly affected in two specific ways by the state of the vegetation in the grassland. Firstly, above-ground vegetation stores part of the water on the grass by intercepting rainfall and condensing dew, effectively regulating and increasing soil moisture. At the same time, the surface vegetation decreases raindrop splash impact, indirectly increasing infiltration capacity. For example, Lin et al. (2015) reported that the soil-water storage capacity of different vegetation types was significantly different at the same site. Secondly, vegetation transpiration contributes to significant losses in soil moisture. The average transpiration in alpine grasslands for our study was 379.35 mm, which was 2.16 times greater than the evaporation from surface soil. Heavy grazing reduced the protection of vegetation and litter; then, we think that the grassland soil is getting drier and drier.

In our study, the lowest amount of soil-water storage occurs in late July or early August, this time period is consistent with the time of maximum transpiration for grassland vegetation. The above-ground biomass was not considered as the main control factor in the BRT model. This was because, although an increase in vegetation can promote plant transpiration, it increases the amount of litter as well. In our study, the quantity of litter was the second key factor (24.02%) influencing grassland water storage. It can be inferred that grass litter regulates the water utilization of grassland by absorbing precipitation and increasing dew condensation, an increase in the quantity of litter will improve the water permeability and retention capacity of soil (Yu et al., 2012). Therefore, the restoration of water conservation in degraded grasslands should begin with the addition of surface litters.

Soil bulk density increased gradually under the trampling action of grazing cattle and sheep (Bilotta et al., 2007), short-term grazing affects the surface soil, resulting in the reduction of soil porosity; therefore, the soil moisture content also decreases gradually with grassland degradation. In addition, the total carbon, organic matter, and total nitrogen, which are closely related to the water holding capacity of grassland, also show a decreasing trend after grassland degradation (Zhao et al., 2007). As a result, the water absorbing matter in soil decreases, and such negative feedback further exacerbates the decline of grassland water conservation function. With different grazing gradients, the soil-water storage is influenced in the following order: lightly grazed grassland > native grassland > moderately grazed grassland > heavily grazed grassland (Guo et al., 2020). Additionally, the adsorption of water by living roots is an important factor affecting grassland soil-water storage. Damage to the root systems in alpine grasslands can result in the decrease of soil moisture content by 22–50% (Chunxia et al., 2007). With the restoration of degraded grassland, the vegetation roots and root exudates gradually increase, as well as the litter and above-ground biomass (Huang et al., 2019). This combination results in an increase in plant residue. During our study, the restoration of the degraded grassland increased grassland soil-water storage by 40.5%. We found that the primary factor influencing the increase of grassland soil-water storage was the quantity of litter, which was caused by an increase in the above-ground biomass. Therefore, it can be inferred that the improvement of water storage in the degraded grassland was mainly due to the improvement of vegetation conditions.

Changes in environmental conditions include global warming, precipitation changes, and soil environment changes, all of which have important consequences to grassland soil-water storage. Air temperature was significantly and negatively correlated with soil-water storage, because higher temperatures increase soil evaporation and transpiration of vegetation, as well as affecting the composition of plant communities. Liu et al. (2018) found that higher temperatures are beneficial to the growth of gramineous plants in alpine grasslands. The transpiration in gramineous plants is obviously higher than that in sedge plants; therefore, temperature increase has a negative influence on soil-water storage. Among all the environmental factors studied, temperature had the greatest effect on grassland water storage. We imply that any increase in temperature will further reduce soil-water storage in the alpine grasslands of the QTP. The average annual temperature has increased by 1.5–2.8°C since 1960 (Wu and Tang, 2017); therefore, further intensification of global warming will have a profound impact on soil-water storage in alpine grasslands.

The direct sources of soil water in alpine grasslands include precipitation, groundwater, and dew. Precipitation is one of the direct sources of soil water, and we found a positive correlation between precipitation and soil-water storage. For example, in the study of Zhang et al. (2017), for every 1 mm increase in precipitation, the soil-water infiltration depth increases by 0.79–1.06 cm. However, precipitation infiltration is not dependent only on precipitation size and duration. In addition, soil moisture, biological crust, and topography play an important role in precipitation infiltration. Heavy grazing causes extensive changes in the grassland soil environment. It will thicken the mattic epipedon of alpine grasslands. The soil biological crusts under heavy grazing will die and become hard, and the soil total porosity will decrease continuously. These effects lead to a reduction in the soil-water holding capacity in alpine grassland. Precipitation infiltration is a complicated biophysiological process affected by a soil type, vegetation, and atmospheric precipitation. In our study, only 7.82% of the variation of soil-water storage was explained by growing season atmospheric precipitation.

Also, wind speed was another main factor affecting grassland soil-water storage, explaining 6.77% of the variation in soil-water storage. Unfortunately, there are very few studies on the effects of wind speed on grassland soil-water storage. Dai et al. (2019) reported that wind speed is the most important factor affecting the groundwater level in the QTP because wind can promote melting ice, snow, and frozen soil that be used to replenish soil moisture. The annual average wind speed in the QTP has considerably decreased over the timescale of our study, which has, in turn, a profound effect on the soil-water storage in the alpine grasslands. Further study is needed to improve our understanding of the relationship between wind speed and soil-water storage.

## Conclusion

We found that restoration of degraded grassland significantly improved grassland vegetation and soil-water storage. The above-ground biomass and soil-water storage increased by 38.4 and 41.9% from severe degradation stage to moderate degradation stage, respectively. Temperature and litters were the most important environmental factors determining grassland soil-water storage. Therefore, for improved grassland management, we suggest that increasing the amount of litter is a good option to promote water recovery in the degraded grassland.

## Data Availability Statement

Publicly available datasets were analyzed in this study. This data can be found at: Data can be applied from Haibei station website: http://hbg.cern.ac.cn/.

## Author Contributions

XG: conceptualization, writing – original draft preparation, and project administration. GC: methodology and resources. QL: validation. LD: formal analysis. DQ: investigation and data curation. YD: writing – review and editing. All authors have read and agreed to the published version of the manuscript.

## Funding

This work was supported by the Natural Science Foundation of China (41730752 and 31700395), the Natural Science Foundation of Qinghai (2020-ZJ-916), and Qinghai innovation platform construction project (2017-ZJ-Y20). The funders had no role in study design, data collection and analysis, decision to publish, or preparation of the manuscript.

## Conflict of Interest

The authors declare that the research was conducted in the absence of any commercial or financial relationships that could be construed as a potential conflict of interest.

## Publisher’s Note

All claims expressed in this article are solely those of the authors and do not necessarily represent those of their affiliated organizations, or those of the publisher, the editors and the reviewers. Any product that may be evaluated in this article, or claim that may be made by its manufacturer, is not guaranteed or endorsed by the publisher.
